# Single event time series analysis in a binary karst catchment evaluated using a groundwater model (Lurbach system, Austria)

**DOI:** 10.1016/j.jhydrol.2014.02.024

**Published:** 2014-04-16

**Authors:** C. Mayaud, T. Wagner, R. Benischke, S. Birk

**Affiliations:** a Institute for Earth Sciences, University of Graz, Heinrichstraße 26, A-8010 Graz, Austria; b Department of Water Resources and Environmental Analytics, Institute for Water, Energy and Sustainability, Joanneum Research Forschungsgesellschaft mbH., Elisabethstraße 18/II, A-8010 Graz, Austria

**Keywords:** Binary karst aquifer, Single event, Overflow behavior, Time series analysis, MODFLOW, Single-continuum model

## Abstract

The Lurbach karst system (Styria, Austria) is drained by two major springs and replenished by both autogenic recharge from the karst massif itself and a sinking stream that originates in low permeable schists (allogenic recharge). Detailed data from two events recorded during a tracer experiment in 2008 demonstrate that an overflow from one of the sub-catchments to the other is activated if the discharge of the main spring exceeds a certain threshold. Time series analysis (autocorrelation and cross-correlation) was applied to examine to what extent the various available methods support the identification of the transient inter-catchment flow observed in this binary karst system. As inter-catchment flow is found to be intermittent, the evaluation was focused on single events. In order to support the interpretation of the results from the time series analysis a simplified groundwater flow model was built using MODFLOW. The groundwater model is based on the current conceptual understanding of the karst system and represents a synthetic karst aquifer for which the same methods were applied. Using the wetting capability package of MODFLOW, the model simulated an overflow similar to what has been observed during the tracer experiment. Various intensities of allogenic recharge were employed to generate synthetic discharge data for the time series analysis. In addition, geometric and hydraulic properties of the karst system were varied in several model scenarios. This approach helps to identify effects of allogenic recharge and aquifer properties in the results from the time series analysis. Comparing the results from the time series analysis of the observed data with those of the synthetic data a good agreement was found. For instance, the cross-correlograms show similar patterns with respect to time lags and maximum cross-correlation coefficients if appropriate hydraulic parameters are assigned to the groundwater model. The comparable behaviors of the real and the synthetic system allow to deduce that similar aquifer properties are relevant in both systems. In particular, the heterogeneity of aquifer parameters appears to be a controlling factor. Moreover, the location of the overflow connecting the sub-catchments of the two springs is found to be of primary importance, regarding the occurrence of inter-catchment flow. This further supports our current understanding of an overflow zone located in the upper part of the Lurbach karst aquifer. Thus, time series analysis of single events can potentially be used to characterize transient inter-catchment flow behavior of karst systems.

## Highlights

• We applied single event auto- and cross-correlation to a karst aquifer.
• We made a simplified MODFLOW model of the area and applied the same methods.
• Results over sampled data suggest that single event analysis is appropriate.
• Numerical results show the importance of overflow location and heterogeneities.
• Both methods suggest an overflow location in the upper part of the aquifer.

## 1 Introduction

Karst aquifers are of primary importance for supplying drinking water to nearly 25% of the world’s population (Ford and Williams, 2007), but their significant reserves are highly vulnerable to contamination and to industrial or intensive agricultural land use. Because karst is a highly heterogeneous environment comprising aperture diameters varying over more than five orders of magnitude (from fracture openings less than 1 mm in the limestone matrix to conduits of more than 10 m width in large caves) there is a need to develop and improve existing tools helping to better understand the processes governing the hydrodynamic behavior of karst systems. As not more than a few percent of a karst aquifer are generally mapped (or explored), it can be defined as grey/black-box system, where the input is routed through to the output without a direct observation of the water transfer. Thus, indirect methods of characterization have been developed to obtain a maximum of information from the karst systems. They are mostly focused on the comparison between the available input and output data and include hydrograph and chemograph analyses using discharge, specific electric conductivity, water temperature, chemical parameters, isotopes and tracer experiments (e.g., Bakalowicz, 2005; Geyer et al., 2013; Kresic and Stevanovic, 2010; Pinault et al., 2001; Rehrl and Birk, 2010).

Time series analysis are signal processing methods belonging to this category, and are mostly used to improve the understanding of the hydrological behavior of karst systems. Mangin (1984) was the first to apply them to the field of karst hydrology. He compared the autocorrelation and power spectral density functions of the discharge of three Pyrenean karst aquifers under the same climatic conditions and deduced that their different responses were due to different degrees of karstification and storage capacities. Larocque et al. (1998) extended the analysis to a broader dataset (piezometric level, water discharge at the inlet and outlet, precipitation, specific electrical conductivity and water temperature) combined with new methods such as cross-correlation, cross-spectral density, coherence function, gain and phase functions, and proved their usefulness for the purpose of water management. Panagopoulos and Lambrakis (2006) applied time series analysis to two Greek karst aquifers well-known for their different karstification and found that the different results were in agreement with the differences in karstification. More recently Bailly-Comte et al. (2008) applied these methods to a small Mediterranean karst system and highlighted the interactions between the karst aquifer and an overflow river. Kovačič (2010) applied autocorrelation, cross-correlation, power spectral density and coherence function to the complex Unica river catchment and improved the understanding of its hydrodynamic behavior, allowing the differentiation of flow paths using two datasets of different time scale. Time series analysis were also successfully used by Amraoui et al. (2003), Bailly-Comte et al. (2011), Bouchaou et al. (2002), Jemcov and Petrič, (2010), Genthon et al. (2005) and Massei et al. (2006) using a broader dataset (rainfall, specific electric conductivity, turbidity, water temperature) and different methods (e.g. spectral and wavelet analyses).

Until now, time series analysis were mostly applied to time-periods covering a period of 1 year or more (Eisenlohr et al., 1997; Kovačič, 2010; Larocque et al., 1998; Mangin, 1984; Panagopoulos and Lambrakis, 2006). Only a few studies (e.g. Bailly-Comte et al., 2008; Budge and Sharp, 2009; Covington et al., 2009, 2012; Valdes et al., 2006) applied the methods at a very short -or single event time scale to provide information about the hydrodynamic behavior of a karst system for short periods. Indeed, as opposed to long-term time series analysis, which is recommended to provide information about the “average” aquifer behavior/properties (Kovačič, 2010; Panagopoulos and Lambrakis, 2006), single event analysis has the potential to show how the system reacts at the scale of a single event only (Bailly-Comte et al., 2008; Covington et al., 2009, 2012; Valdes et al., 2006). This is of primary importance, because karst aquifers are highly dynamic non-linear systems whose behavior may evolve or vary temporarily depending on the hydrological conditions within the system (Mayaud et al., 2013; Wagner et al., 2013).

As demonstrated by the examples cited above, time series analysis has been frequently applied to karst catchments. Although the results from these applications were found to be in qualitative agreement with field observations, it has only rarely been attempted to verify the interpretation more quantitatively by applying the methods to synthetic catchments represented by a numerical model, where aquifer properties and hydrological stresses are known in detail. This approach was followed by Eisenlohr et al. (1997) who evaluated the results from times series analysis using a numerical groundwater flow model. These authors concluded that the results were not only dependent on the system geometry but also on the frequency and type (allogenic vs. autogenic) of the recharge events and on their intensity. In addition, an inappropriate length of the analyzed time series may cause errors in the interpretation. Jeannin and Sauter (1998) concluded that these methods were inappropriate without knowledge of the investigated area to characterize the underground geometry of karst aquifers. Nevertheless, Larocque et al. (2000) applied successfully autocorrelation and cross-correlation analysis to numerical data of a groundwater model representing the Larochefoucault karst aquifer (France). Later, Budge and Sharp (2009) applied short term cross-correlation analysis to a simplified synthetic MODFLOW catchment in order to develop a conceptual understanding of the Barton springs–Edwards aquifer. Their results showed that the cross-correlation was dependent on the input data but also on the geometrical properties of the aquifer.

The purpose of this paper is to improve the interpretation of time series analysis at the scale of single events in karst catchments that are characterized by the existence of a localized recharge component from a sinking stream and by temporarily varying drainage pattern due to the overflow from one spring catchment to another. This involves the need to investigate how physical characteristics of the karst system are reflected in the results from the time series analysis. To this end, two methods, autocorrelation and cross-correlation, are applied to a well investigated field site, the Lurbach karst system (Austria), where an overflow from one sub-catchment to another one is reported by numerous tracer experiments, and to a synthetic karst catchment represented by a numerical groundwater flow model, which accounts for the most relevant features of the field site in a simplified manner.

## 2 Approach

The following subsection 2.1 provides a brief introduction into the two time series analysis methods that are examined in this paper. These methods are evaluated in parallel using both a field site and a synthetic karst catchment represented by a numerical groundwater flow model, which are both described in the subsequent subsections 2.2 and 2.3, respectively. If the time series analysis of the synthetic and the field case yield comparable results, similar aquifer properties and geometries of the overflow section present in the model can be deduced for the Lurbach system.

### 2.1 Methods

#### 2.1.1 Autocorrelation

The autocorrelation function examines how a value depends on the preceding values over a period of time. This function is represented with a correlogram. The slope of the correlogram is determined by the response of the system to an event. If the event has only a short-term influence on the response of the karst system, the slope of the correlogram will decrease steeply and quickly. In contrast, if the system is influenced by an event for a long time, the slope of the correlogram will decrease slowly. Generally the length of the influence of an event is given by the “memory effect” which is according to Mangin (1984) the lag number when *r*(*k*) reaches the value of 0.2. The formula for autocorrelation is (Larocque et al., 1998; Mangin, 1984):

(1) $$r(k)\frac{C(k)}{C(0)}$$

with

(2) $$C(k)=\frac{1}{n}\overset{n-k}{\underset{t=1}{\sum }}(x_{t}-\overset{¯}{x})(x_{t+k}-\overset{¯}{x})$$

where *k* is the time lag and varies from 0 to *m*. According to Mangin (1984) *m* has to be taken as 1/3 of the length of the whole dataset to avoid stability problems.

Applied at a single event or short time scale, autocorrelation has the potential to allow an estimation of the inertia of the system (Valdes et al., 2006). Then, the memory effect shows how the karst conduits react to the event, and cannot be compared to memory effects resulting from analysis of a long time series.

#### 2.1.2 Cross-correlation

Cross-correlation is used to determine the relationship between two variables *x* and *y*. In the case of karst hydrology they are mostly input–output relationships as for discharge–discharge, rainfall–discharge or water level–discharge. The cross-correlation is represented by a cross-correlogram, which has a positive and a negative part. A peak in the positive part means that the input signal has an influence on the output signal. If the cross-correlogram is symmetrical then the two signals respond at the same time. The maximum amplitude and the lag value of the cross-correlogram provide information about the delay which indicates the time of the pressure pulse transfer into the aquifer. If the input signal is a random process, then the cross-correlation function has the form of the impulse response of the system. According to Larocque et al. (1998) the formula for cross-correlation is:

(3) $$r_{\mathrm{xy}}(k)=\frac{C_{\mathrm{xy}}(k)}{\sigma_{x}\sigma_{y}}$$

with

(4) $$C_{\mathrm{xy}}(k)=\frac{1}{n}\overset{n-k}{\underset{t=1}{\sum }}(x_{t}-\overset{¯}{x})(y_{t+k}-\overset{¯}{y})$$

where *σ_x_* and *σ_y_* are the standard deviations of the two time series.

Applied at a single event scale, the cross-correlation shows how the energy is transferred and modified from the input to the output during a flood (Bailly-Comte et al., 2008; Covington et al., 2009) and represents the impulse response of the system.

### 2.2 Field site

**Fig. 1:**
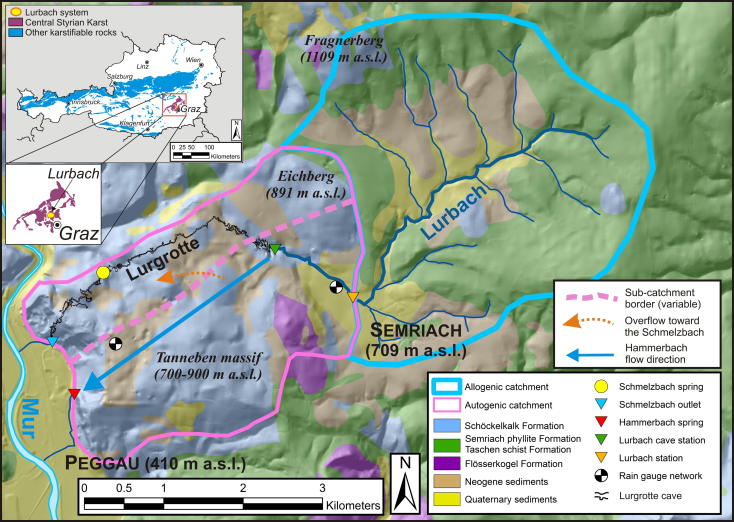
Simplified geological map (modified after Geologische Bundesanstalt, 2005; sheet 164-Graz) of the Lurbach system including approximate flow directions and the monitoring network. The low permeable part (allogenic catchment) corresponds to the topographic catchment whereas the highly karstified part (autogenic catchment) was delineated taking into account results of numerous tracer experiments (Behrens et al., 1992). The boundary between the allogenic and the autogenic units is based on the geological map. The boundary between the Hammerbach and the Schmelzbach sub-catchments is variable depending on the hydrological conditions within the autogenic catchment. Insets: location of the Lurbach system in the Central Styrian Karst and the distribution of karst rocks in Austria (modified after Schubert, 2003).

The area under investigation is a binary karst catchment of 23 km^2^ named Lurbach system, located about 15 km north of Graz (Styria, Austria) and belongs to the Central Styrian Karst (Fig. 1). The upper part of the catchment comprises an area of about 15 km^2^ essentially composed of Paleozoic schists, and is drained by the Lurbach stream in an E–W direction towards the lower part, which is an 8 km^2^ highly karstified unit. After passing the contact schist-limestone, the stream infiltrates along the streambed at a length of some hundred meters and finally disappears into a major sinkhole located right after the entrance of a big cave, the Lurgrotte (entrance at 633 m a.s.l.). Then, the water flows through the conduits and fissures of the limestone massif and resurges at the Schmelzbach outlet and the Hammerbach spring, both located in the valley of the Mur River on the western side of the catchment. The altitude of the whole area ranges between 1.109 m a.s.l. on the top of the Fragnerberg mountain (Fig. 1) and approximately 400 m a.s.l. at the bottom of the Mur valley close to the location of the Hammerbach spring.

The Lurbach system is subject to a climate regime with low winter precipitation (approximately 50 days of snow cover per year) and frequent heavy thunderstorms during the summer (Harum and Stadler, 1992). The mean annual precipitation recorded from 1965 to 2010 at the station of Semriach (Fig. 1) is 880 mm. The maximum precipitation value was recorded at the same station during the summer 1975 with 93.5 mm rain in 1 day.

As it is regularly reported for karst aquifers, the behavior of the Lurbach system varies strongly according to its different hydrological conditions (Harum and Stadler, 1992):

(i) At low and medium water conditions, the allogenic Lurbach waters (mean annual discharge of 141 l/s) supply only the Hammerbach spring (mean annual discharge of 193 l/s). The Schmelzbach outlet drains only autogenic recharge from the limestone massif and has a mean annual discharge of 79 l/s. Thus, both Hammerbach and Schmelzbach sub-systems are then totally separated.
(ii) When the Hammerbach discharge increases above a threshold discharge (about 200 l/s according to Behrens et al., 1992) an overflow from the Hammerbach sub-system to the Schmelzbach sub-system occurs.
(iii) At high water conditions the Lurbach stream can reach a maximum discharge of more than 10 m^3^/s and the whole system is subject to catastrophic flood events. Then, the Lurbach flows directly through the Lurgrotte cave, toward the Schmelzbach, which becomes the main outlet of the karst aquifer and can reach peak discharges up to 10 m^3^/s, whereas the Hammerbach spring shows a limited discharge capacity and cannot drain more than 2 m^3^/s.

The Lurbach karst aquifer has undergone a complex speleological development: on the one hand the Lurgrotte (see Fig. 1) is a well-explored about 3 km long multi-level cave (Wagner et al., 2011). On the other hand, the Hammerbach conduit network is totally unexplored as all attempts to access it failed up to the present. The overflow between the Hammerbach and the Schmelzbach sub-systems is documented only by indirect observation of numerous tracer experiments (Behrens et al., 1992; Kübeck et al., 2013). Moreover, as no boreholes are available in the study area, there is a lack of information concerning the extent and local position of a phreatic zone within the karst massif. The only available information regarding phreatic conditions in the karst aquifer were derived from speleological observations in the cave Lurgrotte itself and by geomorphological observations of several dry caves located in the Tanneben massif (with more than 200 known smaller inactive caves).

The last field campaign in the Lurbach system was conducted from the 28th November 2008 to the 30th December 2008). At the beginning of this time period, a tracer experiment had been carried out (Oswald, 2009). One kilogram of uranine (Uranin AP; AppliChem GmbH, Germany) was continuously injected into the Lurbach stream at the contact between schist and limestone (marked as Lurbach station in Fig. 1) from the 28th November 2008 at 14:11 to the 29th November 2008 00:35. The water levels were recorded at the stations Lurbach cave, Hammerbach spring and Schmelzbach outlet with a frequency of 30 s, whereas the Lurbach station was recorded at a frequency of 5 min. Then, they were converted to discharge rates using control measurements. Precipitation and air temperature were recorded at a 5-min interval at the Ertlhube meteorological station (located at 763 m a.s.l.) on top of the Tanneben karst massif (Fig. 1). During the observation period, two hydrological events occurred. The results from this tracer experiment provide insight into the overflow behavior from the Hammerbach sub-catchment to the Schmelzbach sub-catchment, which will be taken into account when interpreting the results from the time series analysis.

### 2.3 Synthetic karst catchment

In order to evaluate the interpretation of results from single event time series analysis, the groundwater flow model MODFLOW-2005 (Harbaugh, 2005) is used to implement a simplified hypothetical karst setting similar to the Lurbach system. Since the model is applied to a karst setting, it is an obvious idea to employ the Conduit Flow Process (CFP; Shoemaker et al., 2008) for MODFLOW-2005 to account for turbulent flow conditions. However, neither CFP mode 1 (hybrid approach) nor CFP mode 2 (continuum approach) were found to be capable of simulating the rewetting and falling dry of cells representing the transient overflow from the Hammerbach to the Schmelzbach sub-system in the given model setting. Yet, it is important to note that in this work the model is intended to provide general insight into the dependency of the results from the time series analysis on the physical characteristics of a generic type of karst catchment. Thus, similar to the MODFLOW models presented by Ravbar et al. (2011) and Mayaud et al. (2013), the purpose is to represent the natural processes in a simplified manner rather than to provide a quantitative representation of the actual field site. For this purpose, using MODFLOW-2005 without CFP appears to be adequate, as it allows a robust, approximate representation of the overflow dynamics.

The general approach here is to apply single event auto- and cross-correlation to a synthetic catchment and to examine how far the results are similar to or different from those obtained for the Lurbach system. As the model parameters are all known, the differences found in the auto- and cross-correlation of the various scenarios can be clearly attributed to a controlling parameter. Thus, this approach helps to interpret the results from the real system. In addition to a general evaluation of the applied time series analysis methods, this will also improve the current understanding of the Lurbach system.

**Fig. 2:**
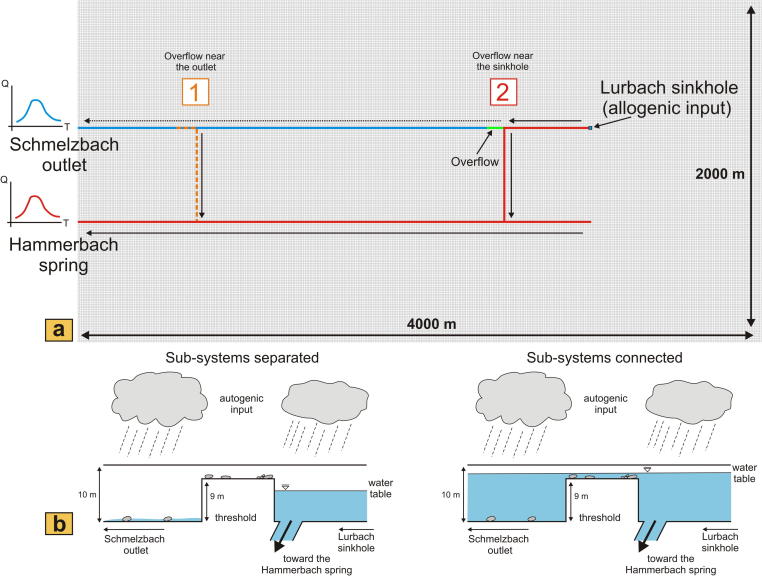
Model setup. (a) Geometry of the MODFLOW model. The allogenic input is implemented using the Well package of MODFLOW in one cell representing the Lurbach sinkhole. The two different assumptions regarding the connection between the Hammerbach sub-catchment and the Schmelzbach sub-catchment are respectively indicated. If an overflow location close to the sinkhole is considered (case 2), the solid arrows indicate the flow toward the Hammerbach when the overflow is inactive, the dotted arrow the autogenic flow toward the Schmelzbach. (b) Schematic illustration of the functioning of the overflow in the MODFLOW model. When the water table is below the level of the threshold both Hammerbach and Schmelzbach sub-systems are separated (left). Then, the Schmelzbach is only supplied by autogenic waters from the limestone massif, whereas the Hammerbach drains all water coming from the sinking Lurbach stream. When the water table rises above the threshold, the overflow is activated and the two sub-systems are connected (right). Then, both Schmelzbach outlet and Hammerbach spring drain allogenic water from the Lurbach sinkhole and share a large part of their respective catchments together.

The model setting is composed of a single unconfined layer with a dimension of 8 km^2^ (4 km × 2 km), which provides a simplified, hypothetical representation of the autogenic part of the Lurbach system (Fig. 2a). The mesh size is set constant to 10 m × 10 m. The Lurbach allogenic input is introduced at a single point (Fig. 2a) using the Well package of MODFLOW, whereas autogenic recharge is given as a constant flux over the whole area. The model was built using the single-continuum approach (Sauter et al., 2006; Teutsch and Sauter, 1991) with two parallel cell lines of high hydraulic conductivity (representing the Schmelzbach and Hammerbach conduits) embedded in a low permeable matrix (see Fig. 2a). The conduit length was set to 3 km for each of the two conduits and is supposed to be close to the real length of the Schmelzbach and Hammerbach network sections (Behrens et al., 1992). The two outlets of the conduits were defined as constant head cells (to simulate the karstic springs) with a two meters difference of relative altitude (the Hammerbach spring being lower than the Schmelzbach outlet) and a linear distance of 550 m to each other. Similarly, the two conduits are connected to each other by a conduit of 550 m length with an overflow located after the first junction (Fig. 2b).

Two different assumptions with regard to the geographical location of the overflow are presented here (Fig. 2a). First, the overflow is located near the Schmelzbach outlet, whereas in the second case it is located close to the Lurbach sinkhole. These two extreme locations were chosen in order to obtain the maximum difference in the hydrological behavior of the springs. The overflow was simulated by elevating a hundred meter stretch of the bottom of the Schmelzbach conduit located just after the connection between the Hammerbach and Schmelzbach sub-systems (Fig. 2b). Within this part of the Schmelzbach conduit, the bottom of the cells was elevated by 9 m relative to the other model cells, such that flow through the conduit occurs only if a threshold water level is exceeded. The initial water table was defined below the overflow level to allow a separation of the two sub-systems at the beginning of the simulation. Then, the Wetting-Capability package of MODFLOW was used to allow rewetting of these cells and thus an activation of the overflow depending on the position of the water table (Fig. 2b). This implies that after a first stress period computed in steady-state the model simulation was transient with 384 stress periods of the same length (30 min), making a total simulation of 192 h. For both locations of the overflow between the two sub-systems, the values of constant head at the two outlets were adjusted such that the hydraulic gradient between the overflow and the Schmelzbach outlet remained approximately equal. First, the Schmelzbach and the Hammerbach catchment were assumed to consist of a homogeneous matrix and conduit system with one value of conductivity (1 m/s) in the conduits, one value in the matrix (10^−5^ m/s) and a constant specific yield for both matrix and conduit (0.01). Then, differences in the hydraulic conductivity and specific yield of the karstic conduits connecting to the two springs inferred from field observations were introduced, to account for heterogeneities of the two sub-systems.

## 3 Results

### 3.1 Field site

**Fig. 3:**
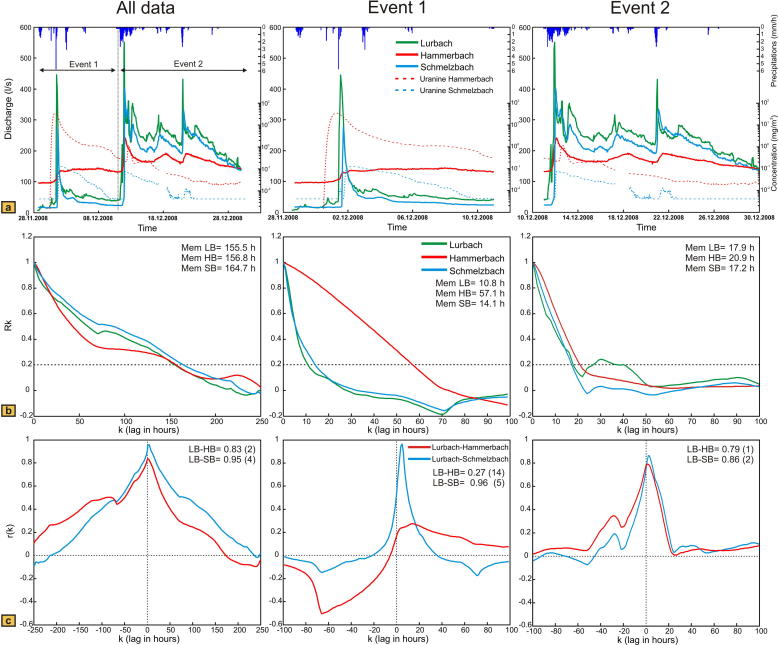
Field data. (a) Discharge recorded at the stations Lurbach cave, Hammerbach spring and Schmelzbach outlet; semi log-scale plot of uranine concentrations at Hammerbach spring and Schmelzbach outlet in December 2008 for the whole period and the two events separated. Vertical bars: hourly precipitation recorded at the Ertlhube rain gauge. (b) Autocorrelation functions and memory effects of the discharge at Lurbach cave (LB), Hammerbach spring (HB) and Schmelzbach outlet (SB) for the whole period and the two events separately; the lags are given in hours. (c) Cross-correlation functions (amplitude and lags) between Lurbach cave–Hammerbach spring (LB–HB) and Lurbach cave–Schmelzbach outlet (LB–SB) for the whole period and the two events separately; the lags are given in hours.

During the one-month period shown in Fig. 3a, two precipitation events of different intensities occurred. The first was short and showed the stronger amplitude (maximum intensity of 5.75 mm per hour at the Ertlhube rain gauge) but the lower cumulative rainfall of 12.9 mm from the 30th November 2008 to the 2nd December 2008; the second had lower intensity (rainfall maximum of 2.6 mm per hour) but extended over a larger time span, resulting in a higher cumulative rainfall of 42.7 mm from the 10th December 2008 to 13th December 2008. Interestingly, no precipitation in form of snow was observed within this period. During these two events the air temperature recorded at the same station stayed mostly between 0 °C and 5 °C, although negatives temperatures were also reported for short periods.

The two precipitation events led to two hydrological events showing different spring responses (the first with a low baseflow, the second with a higher baseflow) recorded at the discharge gauging stations. The raw data of the gauging stations Lurbach cave, Hammerbach spring, Schmelzbach outlet and the precipitation data were transformed in hourly data in order to have an hourly scale for the time series analysis. A gap of approximately 30 h in the discharge dataset from the gauging stations Lurbach cave, Hammerbach spring and Schmelzbach outlet was filled with white noise based on the discharge recorded at Lurbach station which was complete.

The discharge and uranine concentrations show that a large part of the tracer was recorded at the Hammerbach spring a few hours before the first event was recorded at the station Lurbach cave (Fig. 3). Within this time period, no uranine was detected at the Schmelzbach outlet. This indicates that the Lurbach water was drained only toward the Hammerbach spring before the hydrological event. After the beginning of the first event, the tracer was still mainly recovered at the Hammerbach spring, and the breakthrough curve showed an undisturbed tailing. However, some small quantities of uranine were also recorded at the Schmelzbach outlet. This suggests that the overflow towards the Schmelzbach sub-system was activated due to the increasing water table during the event.

An interesting observation is that the overflow seemed to happen at lower discharge than the value of 200 l/s reported in Behrens et al. (1992), which agrees with the findings of Mayaud et al. (2013) and Wagner et al. (2013) that the Hammerbach spring exhibits a changed hydrological behavior since a major flood event happened in 2005. The shapes of the hydrographs further support the assumption of an activation of the overflow from the Hammerbach to the Schmelzbach sub-system because the hydrograph of the Schmelzbach appears to be similar to the hydrograph of the Lurbach, whereas the Hammerbach spring shows a damped response to this event and seems to drain mostly water from the aquifer storage.

Despite the lower maximum intensity of precipitation during the second event, the Hammerbach responds stronger to the resulting recharge pulse possibly due to the higher total amount of precipitation, which leads to an increase of the water table and the hydraulic gradient within the aquifer. The shape of the Schmelzbach hydrograph still appears to be almost identical to that of the Lurbach hydrograph. Uranine is still found in larger quantities at the Hammerbach spring but the concentration recorded at the Schmelzbach outlet responds faster than the Hammerbach to the recharge event. This suggests higher flow velocities and thus probably higher hydraulic conductivity of the Lurbach–Schmelzbach flow path and is in agreement with the assumption that the Schmelzbach still drained most of the Lurbach water during the second event when the overflow was already activated. The higher quantity of tracer recovered at the Hammerbach during the second event might be explained by a remobilization of uranine that was still stored within the Hammerbach sub-catchment down-gradient from the overflow.

Auto- and cross-correlations were computed for the three gauging stations Lurbach cave, Hammerbach spring and Schmelzbach outlet using the time series of the whole period (from the 28th November 2008 to the 30th December 2008 on the left hand side on Fig. 3), and for the two events separately (see Fig. 3 on the middle and right hand side). The two different events were defined taking into account the precipitation distribution and the baseflow discharge rather than the occurrence of discharge peaks. This allows the clear definition of two different periods: a first one with low baseflow (from the 28th November 2008 to the 11th December 2008) followed by a second one with a higher baseflow (from the 11th December 2008 to the 30th December 2008, respectively). As can be seen by comparing the results of auto- and cross-correlation of event 1 and event 2, the system behavior varied strongly during the period. During the first hydrological event, the Hammerbach autocorrelation indicates a damped behavior with a longer memory effect compared to the Lurbach and Schmelzbach autocorrelations. In contrast, the autocorrelation of the Schmelzbach is similar to that of the Lurbach. Correspondingly, the cross-correlation Lurbach cave–Schmelzbach outlet is higher in amplitude and shows a shorter lag time than the cross-correlation Lurbach cave–Hammerbach. This supports the idea that most of the Lurbach event-water was drained towards the Schmelzbach system after the overflow had been activated during the first event. The damped discharge of the Hammerbach is potentially explained by a larger storage capacity within this sub-catchment (Behrens et al., 1992).

When looking at the second event, the results from the time series analysis show a different trend: the Hammerbach autocorrelation function is very similar to those of the Lurbach and Schmelzbach. More specifically, the memory effect apparent in the autocorrelation of Lurbach and Schmelzbach (17.9 and 17.2 h in either case) is still similar to that of the first event (10.8 h and 14.1 h, respectively), whereas in the case of the Hammerbach it is clearly reduced from 57.1 h in the first event to 20.9 h in the second. This suggests that the aquifer storage of the Hammerbach sub-catchment is not able to attenuate the flood pulse of the second event to the same extent as in event 1, possibly because additional conduit pathways are activated at higher water levels (Kübeck et al., 2013). This further indicates that at this time, the Lurbach water pulse is transmitted toward the Hammerbach and Schmelzbach spring at a similar time-scale and that the two sub-catchments have more similar drainage behavior compared to event 1. This is also supported by the finding that the cross-correlation between Lurbach–Hammerbach and Lurbach–Schmelzbach at this time shows the same pattern (*r_xy_* values of 0.79 and 0.86 with lags from 1 to 2 h respectively, opposed to *r_xy_* values of 0.27 and 0.96 with lags of 14 and 5 h during the first event), which shows that the two sub-systems behave similar. Obviously, the different and varying behavior of the Hammerbach and Schmelzbach sub-catchments for these two particular events cannot be inferred from the autocorrelation and cross-correlation of the complete discharge dataset. In particular, the different behavior of Hammerbach and Schmelzbach during event 1 is not apparent if the events are not separated in the analysis (Fig. 3b and c, first column), as both springs then show similar memory effects (156 h for the Hammerbach, 164 h for the Schmelzbach) and similar cross-correlations with the Lurbach (0.83 amplitude and 2 h delay for the Hammerbach opposed to 0.95 in amplitude and 4 h delay for the Schmelzbach). Thus, single event analysis proves useful in this particular example, as it allows distinguishing the change of the global behavior of the Hammerbach sub-catchment during the two events.

### 3.2 Synthetic karst catchment

#### 3.2.1 Homogeneous cases

**Fig. 4:**
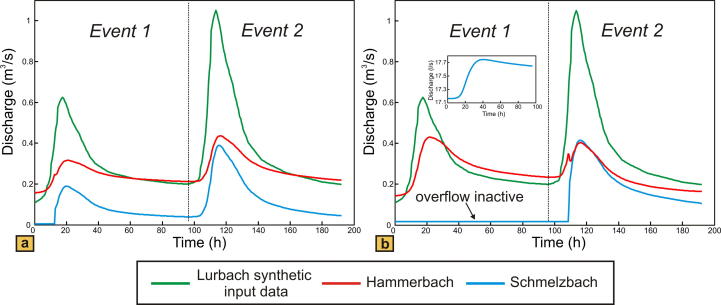
Results from the groundwater flow model: Simulated discharge response of Hammerbach and Schmelzbach due to two recharge events of different intensity. (a) Overflow located close to the Schmelzbach outlet (case 1 in Fig. 2a); (b) overflow located near the Lurbach sinkhole (case 2 in Fig. 2a). The inset shows that some minor matrix flow happens from the Hammerbach sub-catchment to the Schmelzbach sub-catchment during the first event. Hydraulic conductivity, specific yield, and porosity are identical in the two model setups and homogeneous within matrix and conduit system.

Fig. 4 shows the modeling results of the synthetic karst catchment for the overflow located close to the Schmelzbach outlet (case 1 in Fig. 2a) and the overflow located near the Lurbach sinkhole (case 2 in Fig. 2a). The aquifer is unconfined and conduits and matrix are homogeneous, i.e. with the previously mentioned constant values of hydraulic conductivity and specific yield. In order to reproduce a hydrological situation similar to that occurring during the tracer experiment, two artificial allogenic recharge events were defined. The intensity of the allogenic input (Lurbach discharge) was increased from a first to a second event roughly by a factor of two (Fig. 4) in order to see the influence of event intensity on the overflow and discharge characteristics. Autogenic recharge remained constant during the whole simulation.

The comparison of the resulting responses of the synthetic Schmelzbach and Hammerbach hydrographs reveals an activation of the overflow similar to the observation at the field site: in the case of the overflow near the outlet (Fig. 4a) the Schmelzbach hydrograph stays at a constant value over a period of approximately 12.5 h during the first event when the Hammerbach has already started to respond to the Lurbach flood pulse by an increase in discharge. In the case of the overflow near the sinkhole (Fig. 4b), the Schmelzbach does not respond to the first Lurbach flood pulse, and responds later than the Hammerbach during the second event (after 108.5 h). Evidently, the intensity of the first event is too weak to activate the overflow in the case where it is located near the sinkhole (Fig. 4b). Yet, it should be noted that a slight increase in Schmelzbach discharge (see inset in Fig. 4b) is caused by the pressure propagation within the low-permeability matrix flow. It is further noteworthy that the peak discharge of the Schmelzbach slightly exceeds that of the Hammerbach for the second event when the overflow is located near the sinkhole (Fig. 4b), while it stays below in the case with the overflow near the Schmelzbach outlet (Fig. 4a). Likewise the baseflow of the Schmelzbach remains higher with the overflow near the sinkhole than with the overflow near the outlet. These observations are explained by the size of the autogenic sub-catchment of the Schmelzbach, which increases with increasing distance of the overflow from the outlet. Thus, the location of the overflow in the groundwater model is found to be of primary importance.

**Fig. 5:**
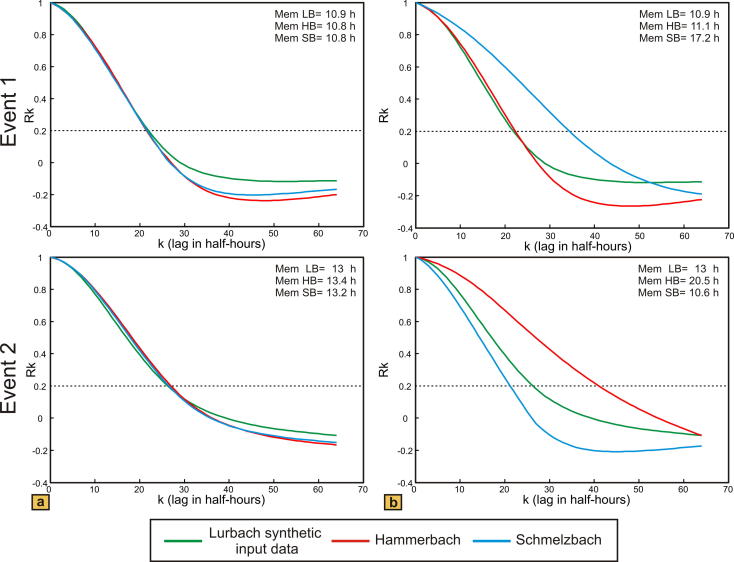
Autocorrelation functions of the simulated Lurbach (LB), Hammerbach (HB) and Schmelzbach (SB) discharges presented in Fig. 4 for the two separated events; (a) overflow near the outlet, (b) overflow near the sinkhole. Here the lags are given in half hours. Memory effects are given in hours.

The autocorrelation of the hydrographs of the two models is presented in Fig. 5. With the overflow located near the outlet (Fig. 5a) the values of the memory effect for the Hammerbach and Schmelzbach are identical for both events and the values obtained for the first and the second event are similar (approximately 10.8 h during the first event; nearly 13 h during the second event). Contrary, the model with the overflow located near the sinkhole (Fig. 5b) shows different memory effects for Hammerbach and Schmelzbach and the memory effect is found to be different for the two events. The result from the first event can be explained by the inactivity of the overflow: almost all Lurbach allogenic water is drained towards the Hammerbach spring, whereas the Schmelzbach outlet is supplied only by a constant flux of autogenic water and a small amount of Lurbach water transferred through the matrix (see Inset in Fig. 4b). As a consequence, the memory effect for the Schmelzbach is higher than that for the Hammerbach. During the second event both Hammerbach and Schmelzbach are supplied by allogenic water from the Lurbach but still have different memory effects (11.1 h during the first event and 20.5 h during the second for the Hammerbach; 17.2 h during the first event and 10.6 h during the second for the Schmelzbach). As opposed to the first event the Schmelzbach responds faster (lower memory effect) than the Hammerbach. This is due to the sudden activation of the overflow forcing a high proportion of Lurbach water to flow towards the Schmelzbach system. Similarly, the memory effect of the real Schmelzbach was found to be lower than that of the real Hammerbach when the overflow was activated in the first event observed at the field site (see event 1 in Fig. 3b). The above findings thus demonstrate that the memory effect is influenced by the overflow. Yet, it should be noted that differences in the memory effects of different events can also be caused by different input signals, which suggests that the interpretation of the memory effect in a setting with strongly varying input signals is not straightforward and should be supported by additional evidence from other methods.

**Fig. 6:**
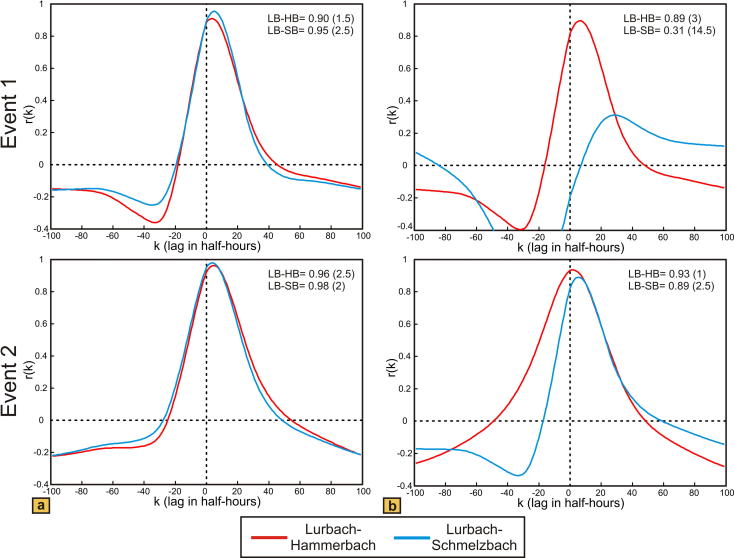
Cross-correlation functions of the simulated Lurbach–Hammerbach (LB–HB) and Lurbach–Schmelzbach (LB–SB) discharges presented in Fig. 4 for the two separated events; (a) overflow near the outlet, (b) overflow near the sinkhole. Here the lags are given in half hours. Cross-correlation results are given in hours.

Single event cross-correlation results obtained with the two model set-ups are shown in Fig. 6. Corresponding to the results from the autocorrelation, the Lurbach–Hammerbach and Lurbach–Schmelzbach cross-correlation functions are similar in both events (more than 0.90 in amplitude for all cases) if the overflow is located near the outlet (Fig. 6a). In contrast, the model with the overflow near the sinkhole (Fig. 6b) shows a lower cross-correlation amplitude for the Lurbach–Schmelzbach during the first event when the overflow is not activated and a higher amplitude reaching almost the Lurbach–Hammerbach cross-correlation during the second event. Yet even during the second event the amplitude of the Lurbach–Hammerbach cross-correlation is higher than that of the Lurbach–Schmelzbach cross-correlation. In addition, the maximum of the lag time of the Lurbach–Schmelzbach cross-correlation is larger than that of the Lurbach–Hammerbach cross-correlation (14.5 h opposed to 3 h during the first event and 2.5 h opposed to 1 h during the second event). These findings are explained by the activation of the overflow during the second event, which causes a delayed response of the Schmelzbach relative to the Hammerbach. This is in striking contrast to the observation at the field site, where the real Lurbach–Schmelzbach cross-correlation exhibits a higher amplitude and lower lag time than the real Lurbach–Hammerbach cross-correlation when the overflow is activated (see event 1 in Fig. 3c). Thus, the autocorrelation of the observed hydrograph and that obtained with the model where the overflow is close to the sinkhole show some similarity (memory effect of Schmelzbach lower than that of Hammerbach when the overflow is activated), but the cross-correlation from the first event at the field site clearly suggests a more attenuated and damped response of the Hammerbach than that obtained with the model. In the above considered model scenarios the matrix and the conduit system were assumed to be homogeneous. As karst aquifers are highly heterogeneous an important question is how heterogeneities may influence the numerical spring response and the resulting shape of the auto- and cross-correlation functions. This is considered in the following sub-section.

#### 3.2.2 Heterogeneous cases

As the Schmelzbach system is assumed to be higher karstified than the Hammerbach system (Behrens et al., 1992), heterogeneities were introduced in the groundwater model by increasing the conduit hydraulic conductivity from 1 m/s to 1.2 m/s from the overflow location to the Schmelzbach outlet. Moreover, as the Hammerbach is reported to have the higher aquifer storage (Behrens et al., 1992), the value of specific yield of the conduit cells within this sub-catchment was consequently increased from 0.01 to 0.5.

**Fig. 7:**
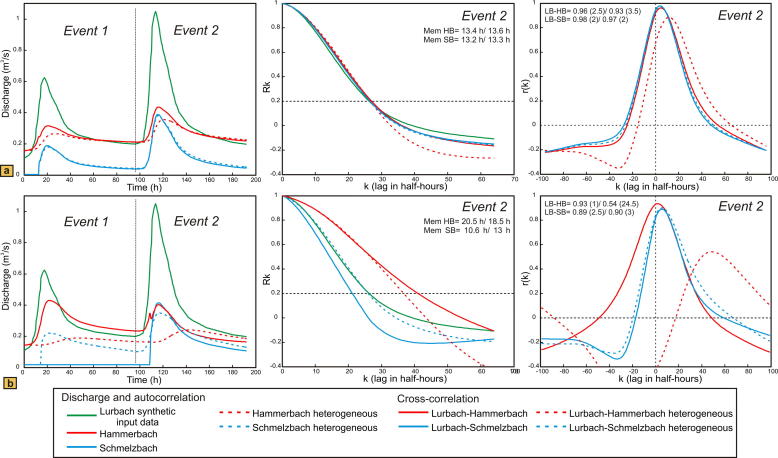
Comparison of discharge, auto- and cross-correlation from the homogeneous model (solid lines) and the heterogeneous one (dashed lines); (a) overflow near the outlet, (b) overflow near the sinkhole. While matrix and conduit parameters of the Schmelzbach and Hammerbach sub-catchments are identical in the homogeneous model, the hydraulic conductivity of the Schmelzbach conduit and the specific yield in the Hammerbach conduit were increased in the heterogeneous model. Auto- and cross-correlation are shown for the second event only. Here the lags are given in half hours. Hourly auto- and cross-correlation results are indicated for the homogeneous and the heterogeneous model, respectively.

Fig. 7 presents a comparison of hydrographs, auto- and cross-correlation functions between the heterogeneous case (dashed lines) and the homogeneous one (solid lines) for the overflow located near the outlet (Fig. 7a) and the overflow located near the sinkhole (Fig. 7b). Auto- and cross-correlation are shown only for the second event. For both geometries the Hammerbach hydrograph is evidently more damped in the heterogeneous than in the homogeneous case. In addition, the Hammerbach response in the heterogeneous model appears to be delayed compared to the homogeneous case if the overflow is located near the sinkhole (but not when the overflow is near the outlet). Another important result in the heterogeneous model with the overflow near the sinkhole is the activation of the overflow during the first event (after 14 h) whereas it remained inactive for the homogeneous case. This result is consistent with the lower value of 12.5 h found for the overflow located near the outlet.

The autocorrelation for the model with the overflow located near the outlet shows almost no influence of the heterogeneities and yields similar memory effects for Hammerbach and Schmelzbach than those of the homogeneous case. Contrary, the model with an overflow located near the sinkhole shows different memory effects for the Hammerbach and the Schmelzbach and the values differ from those of the homogenous case (18.5 and 13 h as opposed to 20.5 and 10.6 h for the homogeneous case).

The results of the cross-correlation analysis correspond to those of the autocorrelation: the heterogeneous model with the overflow located near the outlet has cross-correlograms similar (Lurbach–Schmelzbach) or slightly damped and delayed (Lurbach–Hammerbach) compared to the homogeneous case. Evidently, the heterogeneity has little influence on the spring response if the overflow is close to the outlet. In this case the greater part of the aquifer is upstream of the overflow and thus shared by the two springs, while there is only a small part downstream of the overflow with two separate heterogeneous sub-catchments. In contrast, the model with the overflow located near the sinkhole is strongly influenced by the heterogeneities: the Lurbach–Hammerbach cross-correlation is clearly damped, whereas the Lurbach–Schmelzbach cross-correlation is only slightly changed. Thus, the Schmelzbach is the main outlet of the system and has a more flashy behavior than the Hammerbach, which corresponds well with the field observations and further supports a location of the overflow in the upper part of the aquifer.

Results from the aforementioned tracer experiment (Fig. 3a) also suggest an overflow location close to the sinkhole: since most of the tracer was recovered at the Hammerbach spring and even in the time period when the overflow was active only a small amount of tracer was detected in the Schmelzbach, the overwhelming majority of tracer must have passed the overflow location at the time when the overflow became active. In agreement with the results of the single event time series analysis these findings support an overflow location within the upper part of the Lurbach aquifer.

## 4 Conclusion

Single event time series analysis was combined with a groundwater model to examine how the inter-catchment flow in a karst aquifer varies during a period of 1 month and how this is reflected in the results from the time series analysis. Auto- and cross-correlation of the observed data differ for the two events considered here, thus showing the necessity to make a single event analysis rather than an analysis of the whole dataset. The numerical model implemented with MODFLOW was able to reproduce the general overflow behavior observed in the Lurbach system. Results of auto- and cross-correlation of the numerical model showed that aquifer heterogeneities and the overflow location were of primary importance. The model with an overflow located near the outlet was found to be relatively insensitive to a variation of hydraulic parameters. Contrary, the model with an overflow located near the sinkhole showed a high sensitivity to heterogeneities and was selected as the most probable option to better reproduce the observed behavior of the Lurbach system. Results from the model with the overflow near the sinkhole and heterogeneous aquifer parameters were found to be in good agreement with the field observations. Thus, in agreement with evidence from tracer tests an overflow location in the upper part of the aquifer rather than in the lower part is suggested. In summary, single event time series analysis was found to be useful for characterizing transient inter-catchment flow and aquifer properties (overflow location, aquifer heterogeneity) controlling the spring responses to recharge in this karst catchment. Yet, it is important to note that results from time series analysis need to be complemented by other aquifer characterization techniques to interpret them in terms of flow processes and aquifer properties. In this work, tracer testing and groundwater modeling proved useful for this purpose.
